# On the Interaction of *Clostridium perfringens* Enterotoxin with Claudins

**DOI:** 10.3390/toxins2061336

**Published:** 2010-06-08

**Authors:** Anna Veshnyakova, Jonas Protze, Jan Rossa, Ingolf E. Blasig, Gerd Krause, Joerg Piontek

**Affiliations:** Leibniz-Institut für molekulare Pharmakologie, Robert-Rössle-Str.10, 13125 Berlin, Germany; Email: veshnyakova@fmp-berlin.de (A.V.); protze@fmp-berlin.de (J.P.); rossa@fmp-berlin.de (J.R.); iblasig@fmp-berlin.de (I.E.B.); gkrause@fmp-berlin.de (G.K.)

**Keywords:** Claudins, *Clostridium perfringens* enterotoxin, drug delivery, tight junction

## Abstract

*Clostridium perfringens* causes one of the most common foodborne illnesses, which is largely mediated by the *Clostridium perfringens* enterotoxin (CPE). The toxin consists of two functional domains. The N-terminal region mediates the cytotoxic effect through pore formation in the plasma membrane of the mammalian host cell. The C-terminal region (cCPE) binds to the second extracellular loop of a subset of claudins. Claudin-3 and claudin-4 have been shown to be receptors for CPE with very high affinity. The toxin binds with weak affinity to claudin-1 and -2 but contribution of these weak binding claudins to CPE-mediated disease is questionable. cCPE is not cytotoxic, however, it is a potent modulator of tight junctions. This review describes recent progress in the molecular characterization of the cCPE-claudin interaction using mutagenesis, *in vitro* binding assays and permeation studies. The results promote the development of recombinant cCPE-proteins and CPE-based peptidomimetics to modulate tight junctions for improved drug delivery or to treat tumors overexpressing claudins.

## 1. Introduction

*Clostridium perfringens* enterotoxin (CPE) causes the gastrointestinal symptoms of *C. perfringens* type A food poisoning, which is one of the most common foodborne illnesses in the USA and Europe. In addition, CPE is thought to contribute to diarrhea that is associated with antibiotic treatment, and to cause gastrointestinal illness frequently in domestic animals [1,2]. CPE binds to transmembrane proteins on human ileal epithelium [3]. These CPE-receptors are claudin-3, -4 (Cld3, -4) and some other members of the claudin family, which are tight junction (TJ) proteins. After binding, pore formation in the plasma membrane of the host mucosa cell leads to fluid and electrolyte loss along with epithelial cell death and the known clinical symptoms of CPE-intoxication [3].

A C-terminal fragment of CPE (cCPE) is not cytotoxic but still binds to certain claudins and modulates the function of TJ formed by claudins mainly. Treatment of epithelial monolayers with non-cytotoxic cCPE increases paracellular permeability [4] and enhances drug absorption in rat jejunum 400-fold relative to sodium caprate, which is in clinical use [5]. cCPE removes its receptor—Cld4—specifically from tight junctions while distribution of those claudins that do not bind cCPE are unaffected [4]. The cCPE-induced subtype-specific removal of claudins, and therefore the opening of TJ, is mediated by a different mechanism than cytotoxicity of full length CPE. This is concluded because the opening is slow, reversible and does not affect integrity of the plasma membrane, while the toxicity is caused by fast and irreversible increase in membrane permeability. These findings have implicated cCPE as a pharmacological tool (i) to modulate tight junctions to improve drug delivery across tissue barriers and (ii) for treatment of tumors overexpressing claudins. In this review, the *Clostridium perfringens* enterotoxin and its cellular receptors (defined claudin subtypes) are described. Here, the focus is a summary of recent progress in molecular characterization of the cCPE-claudin interaction that can be used for an improved design of CPE-based modulators of claudins.

## 2. *Clostridium perfringens* and Its Toxins

The gram-positive bacterium *Clostridium perfringens* is a human and veterinary pathogen [6]. *C. perfringens* produces at least 14 different protein toxins [6]. Alpha toxin, produced by all toxinotypes of *C. perfringens*, has hemolytic and lethal activities [7]. It is the primary mediator of gas gangrene caused by *C. perfringens* [8]. Toxinotypes B and C produce pore-forming beta toxin [9], shown to be lethal in a mouse model [10]. Types B and D express epsilon toxin, which is also a pore forming toxin [11,12,13]. Type E of *C. perfringens* produces iota toxin with ADP-ribosyltransferase activity.

Additionally, CPE is deemed to be the *C. perfringens* toxin that is most relevant for pathologic effects in human intestines. Unlike the other toxins, CPE is produced only by sporulating cells and accumulates in a large inclusion body inside the mother cell, from which it is released after lysis at the end of sporulation [14,15]. It causes the symptoms of *C. perfringens* type A food poisoning and those of non-foodborne gastrointestinal illnesses. A correlation between food poisoning and *C. perfringens* was first established in the 40s and 50s of the 20th century. The poisoning effect was demonstrated after development of the rabbit ileal loop model in 1968 [16] and the ability to cause diarrhea in humans was shown in 1971 [17]. Later on, purified CPE was confirmed to be responsible for diarrhea in animal models [18] and humans [19]. In addition, knock-out mutants confirmed CPE to be the toxin responsible for disease caused by CPE-positive strains [20]. 

## 3. *Clostridium perfringens* Enterotoxin (CPE)

CPE is a single polypeptide with 319 amino acids and a molecular mass of 35 kDa [21]. Significant homology to any other bacterial toxins is neither recognized on the DNA nor on the protein level. However, on the amino acid level, it has 27% identity and 46% similarity to the non-toxic hemagglutinin components of the type *C. botulinum* toxin [22].

Intoxication by CPE causes, firstly, inhibition of absorption of ions and fluid by intestinal epithelial cells and, secondly, death of these cells leading to secretion of fluid into the intestinal lumen [23]. The pathophysiological and cellular effects of CPE were studied in animal models, such as rat and rabbit ileum [24], and sections of human intestine were examined *ex vivo* [3] and *in vitro* with cell lines, e.g., Vero cells and human colon carcinoma cells Caco-2 cells [25]. The Caco-2 cells have the advantage that they show characteristics of enterocytes since they form complete TJ and microvilli brush borders like native intestinal epithelial cells. Using Caco-2 cells grown on Transwell® permeable supports, it could be shown that CPE-incubation is 2–3 times more effective after basolateral application in comparison to the apical application [25]. CPE-incubation leads to the lysis of cultured epithelial cells from intestine, liver and kidney [26,27].

The first step of CPE-induced toxicity is the binding of CPE to its receptors on the surface of CPE-sensitive cells. The TJ proteins Cld3 and Cld4 have been identified to be cellular receptors for CPE [28]. After association of CPE with its receptors, a 90 kDa SDS(sodium dodecylsulfate)-sensitive “small complex”, containing CPE and claudins is formed [29,30]. Oligomerization of the “small complex” is proposed to result in formation of a 155 kDa SDS-resistant “large complex” [28,29,30]. This leads to a massive increase in membrane permeability for molecules <200 Da. It was suggested that CPE - as part of the 155 kDa complex - partially integrates in the plasma membrane, thereby leading to formation of a transmembrane pore [31,32]. The Ca^2+^ influx through this CPE-pore results in cell damage. It was proposed that this damage provides CPE access to TJ and leads to formation of a 200 kDa complex containing occludin and to internalization of TJ proteins. TJ damage and cell death by apoptotic and oncotic processes increase paracellular permeability and thereby contribute to the diarrhea of CPE-induced gastrointestinal disease [25,33]. Using gel shift assays and size exclusion chromatography, the 155 kDa complex was resized as ~425–500 kDa and suggested to consist of 12 claudin and six CPE molecules. The 200 kDa complex was resized as a ~550–660 kDa complex [29]. 

### 3.1. Functional domains of CPE

Two major domains have been functionally distinguished in CPE: the N-terminal half shares several structural similarities with ß-poreforming toxins [32] and mediates the cytotoxicity, whereas the C-terminal half contains the claudin-binding domain [34]. Analysis with recombinant deletion mutants of CPE revealed the region meditating the different activities of CPE in more detail. The 44 N-terminal amino acids have an inhibitory effect on the cytotoxicity [34]. The region 45–167 is involved in membrane insertion as well as “large complex”- and pore-formation. More precisely, a specific region between D45 and S59 is required for the formation of the “large complex” [2,34,35]. The region 81 to 106 is essential for pore formation [32].

### 3.2. C-terminal claudin-binding domain of CPE

The C-terminal region 290–319 is sufficient for binding to claudins, as was shown with corresponding recombinant C-terminal CPE-fragments and respective synthetic peptides [36]. Removal of these 30 C-terminal amino acids inhibited binding to claudins completely [34]. However, longer CPE-fragments interact much stronger with claudins than cCPE_290–319_ [37]. Van Itallie and colleagues solved the crystal structure of cCPE_194–319_, which most likely contains the complete claudin-binding domain [38]. This domain comprises the residues A205–F319 and forms a nine-strand β-sandwich. The core claudin-binding region—S290–F319—is formed by strands β8 and β9, which are located in the center of the opposing beta sheets, and the surface loop between these strands. Consequently, peptides restricted to the amino acids 290–319 are not expected to have the same conformation as the corresponding region in the full beta sandwich. This explains, at least partly, why cCPE_290–319_ binds more weakly to claudins than longer cCPE-fragments [37].

The structure of cCPE194–319 shows similarities to the receptor-binding domains of other bacterial toxins [38]. This was unexpected, since no strong homology to these proteins is given at the amino acid sequence level. However, similarities in the 3D structure, e.g., to those domains within the ColG collagenase of *Clostridium histolyticum* [39] and the Cry4Ba toxin of *Bacillus thuringiensis* [40], suggest a common evolutionary origin.

## 4. The CPE-Receptors: Claudins

The cellular receptors for CPE were discovered by the pioneering work of Katahira *et al.* [41]. In their expression cloning approach, a cDNA library from CPE-sensitive Vero cells was introduced in CPE-insensitive fibroblasts. The transfectants were incubated with recombinant cCPE and those expressing a CPE-receptor sorted by fluorescence activated cell sorting (FACS). The transfected plasmids were recovered, sequenced and the ability of the encoded protein to bind CPE was verified. This resulted in identification of the CPE-receptor, CPE-R [41]. Further sequence analysis and screening revealed that the androgen withdrawal apoptosis protein (RVP1) is structurally related to CPE-R and an additional receptor for CPE, CPE-R2 [28]. Later, both were characterized as members of the claudin (Cld) protein family, namely Cld4 and Cld3 [42]. Other family members, e.g., Cld1 or -2 were originally described not to bind to CPE: fibroblasts transfected with Cld1 or -2 did not show clear CPE-binding nor CPE-induced cell death [4,43]. However, recently, both have been demonstrated to bind with weak affinity to CPE (see below and [44]). To date, 24 subtypes of claudin were identified in mammalian cells. They are known as tetraspan membrane proteins with two extracellular loops (ECLs).

Claudins form the backbone [45] of TJ, cell-cell junctions in the apical part of the lateral plasma membrane in epi- and endothelia. TJ form a belt-like seal around each cell that limits and regulates paracellular permeability and that is essential for tissue barriers. TJ appear as fusions of the membranes of two neighboring cells (transmission electron microscopy) and as a continuous network of strands of transmembrane proteins [46]. Occludin [47], tricellulin [48] and marvelD3 [49] are other tetraspan transmembrane TJ proteins. Scaffolding proteins like zonula occludens proteins (ZO1–3) and signaling proteins are associated with TJ as, for instance, by binding of their PDZ-domains to respective binding motifs at the C-terminus of claudins [45]. These membrane associated proteins regulate assembly and disassembly of TJ [50,51]. Claudins are known to tighten the paracellular cleft size- and charge-selectively and can be functionally divided in barrier-forming, e.g., Cld1, -3, -4, -5, and pore-forming claudins, e.g., Cld2, -7, -10, -16 [45]. Expression of barrier-forming claudins decreases paracellular permeability of ions, solutes and proteins in a subtype-specific manner. In contrast, pore-forming claudins increase paracellular permeability for ions, e.g., Cld2 for monovalent cations. The tissue-specific combination of claudin subtypes expressed in a given tissue is assumed to determine the permeability properties of the TJ [45]. Residues in the first extracellular loop (ECL1) of claudins determine the ability to form paracellular ion pores as well as their charge selectivity [52].

Claudins copolymerize in heteropolymers by homo- and heterophilic interactions between the subtypes by different modes of action. c*is*-interaction (between molecules along one membrane) and *trans*-interaction (between molecules of two opposing cell membranes) contribute to the formation of polymeric intramembranous TJ strands [45,53]. The pattern of heterophilic compatibility of different claudin-subtypes is just partly known. However, assays applying cellular reconstitution of TJ-strands, confocal analysis of subcellular distribution and FRET (fluorescence resonance erergy transfer) of claudins as well as electron microscopy are used to investigate pattern and mechanism of claudin polymerization in more detail [53,54]. 

On the amino acid sequence level, two subgroups of claudins can be distinguished. Classic claudins (Cld1 up to -10, -14, -15, -17, -19) share higher homology among each other than non-classic claudins (Cld11, -12, -13, -16, -18, -20 up to -24) [45]. Classic claudins are likely to share a common helix-turn-helix structure of the ECL2 that is involved in paracellular tightening [54,55,56]. Since the structure of claudins is unknown, a molecular model of the ECL2 was generated by combination of cell biological analysis of claudin mutants and bioinformatics homology modeling [45,54,57]. As an example, for classic claudins, a model of Cld5 based on high sequence homology to the protein BB2244 from *Bordetella bronchiseptica* and the corresponding crystal Structure (2BDV) was developed. This model was also consistent with experimental data, since it could explain folding defects of the Cld-5-ECL2 mutants analyzed. This model predicted a helix-turn-helix structure. Furthermore, a model of an ECL2-*trans*-dimer of Cld5 was derived from experimental data demonstrating that defined residues in the ECL2 are essential for *trans*-interaction of the protein [54]. In particular, aromatic residues of two opposing ECL2 form an aromatic core which is necessary for *trans*-interaction. Strikingly, the residues suggested to be involved in folding and in *trans*-interaction, are highly conserved among classic claudins [45]. This supports the assumption that the structure of the ECL2 and the mechanism of *trans*-interaction is, at least, partly similar for all classic claudins. 

**Figure 1:**
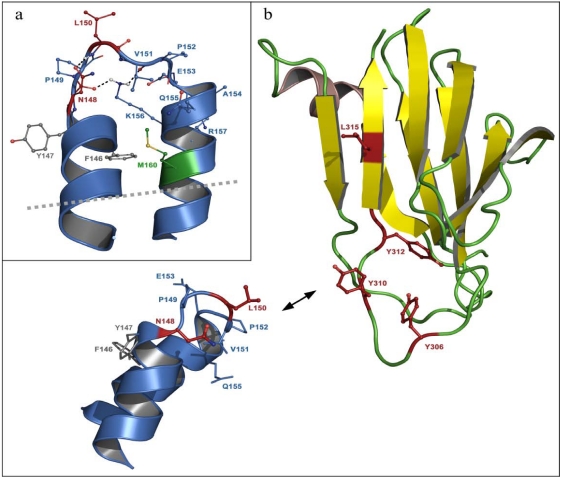
(**a**) Homologous helix-turn-helix model of the ECL2 of mouse Cld3 based on the fragment of PDB code 2BDV in front view. Residues F146 to R157 and M160 are shown (ball and stick), key residues for CPE-claudin interaction N148 and L150 [37] are given in red. M160 (green) was also reported to be involved in CPE-claudin interaction [44]. Residues F146, Y147 (grey), and R157 (blue) correspond to aromatic residues F147, Y148, and Y158 in Cld5, which were found to be important for *trans*–interaction. Heteroatoms are red (oxygen), dark blue (nitrogen), white (hydrogen), and yellow (sulfur). Black dashed lines: hydrogen bonds, grey dashed line: cell-membrane. (**b**) Supposed spatial regions of interaction between binding sensitive residues (Y306, Y310, Y312, and L315 from literature [60]) visualized at the CPE194–319 x-ray structure (PDB code 2QUO) and residues (148NPLVP, Q155) in Cld3. Key-residues for the CPE-claudin interaction are highlighted in red.

In particular, experimental data and modeling suggested a helix-turn-helix structure for the ECL2 of the CPE-binding Cld3 (Figure 1 and [37]), similar to the one of Cld5 [54]. Since CPE binds to the ECL2 of Cld3 [43], the molecular model of the Cld3-ECL2 can be used to analyze the CPE-claudin interaction in more detail [37]. 

## 5. The Interaction between *Clostridium perfringens* Enterotoxin and Claudins

### 5.1. Methods used to investigate interaction between CPE and claudins

To investigate the interaction between CPE and claudins different methods have been applied. These are described and compared in the following sections.

#### 5.1.1. Use of peptides in arrays and surface plasmon resonance spectroscopy

Synthetic peptides have been used to investigate CPE-claudin interactions. This is a useful strategy since the minimal claudin binding region of CPE as well as the CPE-binding ECL2 of claudins are relatively short, 30 and 15–25 amino acids, respectively (for details see Chapters 5.2 and 5.3). Arrays of immobilized 15–20 mer ECL2 peptides where incubated with recombinant GST-CPE fusion proteins to identify residues in the ECL2 responsible for CPE-binding [37]. For quantitative analysis of binding surface plasmon resonance spectroscopy (SPR) ECL2 peptides were immobilized on biacore sensor chips and superfused with recombinant GST-CPE fusion proteins. Similarly, CPE_290–319_ peptides were immobilized and superfused with recombinant Cld4 proteins to define structural constraints in Cld4 for CPE-binding [58]. 

However, peptides often do not have the native conformation as it occurs in the full length protein. This is of particular importance for claudins, since the short ECL2 is forced to form a loop structure by the adjacent transmembrane domains. Similarly, CPE_290–319_ peptides cannot form the native binding domain, due to missing essential β-strands [37,38]. Hence, results obtained with peptides have to be verified by assays using full length proteins or constructs shown to form the relevant domains. 

#### 5.1.2. Pull-down assays

Pull-down assays are widely used for analysis of interaction between cCPE and full length claudins. These assays can be performed in different ways. (i) In some studies, lysates of cells expressing full-length Cld4 were incubated either with His-tagged cCPE-wt or cCPE-mutant, and then mixed with Ni-resin beads. Bound proteins were analyzed by SDS-PAGE (polyacrylamide gel electrophoresis) followed by Western blot [59,60]. (ii) Other authors use tagged claudins—Van Itallie *et al.* reported using His-tagged Cld4—to immobilize protein molecules on beads and afterwards to incubate the beads with purified cCPE. Eluted Cld4–cCPE complexes were further resolved by SDS-PAGE and detected by Coomassie Brilliant blue staining [38]. (iii) GST-tagged cCPE bound to gluthatione-sepharose beads was incubated with human embryonic kidney (HEK)-293 cells, transiently transfected with Cld3. Eluted cCPE–Cld3 complexes and unbound fractions were analyzed by means of SDS-PAGE and Western-blot [37]. In addition, pull-down assays were performed with lysates of cells expressing endogenous claudins [60,61]. 

Pull-down assays have the disadvantage to depend on detergent solubilization of the transmembrane claudin proteins. It has to be considered that detergents can strongly alter the conformation of transmembrane proteins and, thereby, change the interaction properties of proteins. Moreover, compared to binding assays with purified recombinant proteins or peptides (see Chapter 5.1.1), one cannot exclude interference by other proteins.

#### 5.1.3. Cellular binding assays

Cellular binding assays, in comparison to pull-down assay, allow investigation interaction of cCPE with full length claudins in their native membrane environment. Either cells expressing endogenous claudins, like Caco2 cells, or claudin-transfected cells can be used. Cells with endogenous claudins and functional tight junctions have the advantage that they form a physiologically relevant cellular barrier. However, claudin-transfection of cells without endogenous tight junction proteins enables the analysis of individual claudins without interference of other tight junction proteins.

Claudin-expressing cells are incubated with CPE-constructs, washed, lysed and the lysates are resolved by SDS-PAGE and analyzed by Western blot for the amount of bound CPE and expressed claudins [37] Alternatively, CPE-binding can be detected by measuring the radioactivity of I^125^-labeled CPE [41]. 

The following assays are also based on the CPE-binding to cellular claudins:

##### 5.1.3.1. Assay of CPE cytotoxicity

The cytotoxic effect of CPE-constructs is used to measure their activity [62]. Either full-length CPE or cCPE fused to a toxic effector domain of another protein, e.g., synthesis inhibitory factor (PSIF), is incubated with cells expressing claudins of interest. These are either cells expressing endogenous claudins such as Caco-2 and Marbin Darby canine kidney (MDCK) cells, or claudin-transfected HEK293 and L-cells, both without endogenous claudins. The cell death as a measure for CPE-binding is determined by assessing cell membrane integrity. One common way is to evaluate the release of lactate dehydrogenase (LDH) from dead cells to the culture medium [59,60,63].

##### 5.1.3.2. Competition binding assay

A modification of the method mentioned above uses competition of binding partners. For instance, the cytotoxic cCPE_wt_-PSIF competes with non-toxic cCPE-constructs for binding to claudin-expressing cells [59,60]: the better the non-toxic cCPE-construct interacts with its receptor, the stronger the inhibition of cCPE_wt_-PSIF binding and the lower the cytotoxic readout. In this assay, cells are pretreated with the cCPE-construct of interest then incubated with cCPE_wt_-PSIF, and afterwards, cell death is measured by LDH release.

In another approach, different claudin-constructs compete for binding to cCPE [62]. In this assay, cCPE is pretreated with solubilized recombinant claudin proteins before it is applied to cells expressing native claudins. 

#### 5.1.4. Methods for investigation of cCPE effects on paracellular permeability

Since it is well known that the CPE-receptors (claudins) constitute the backbones of TJ in membranes of epithelial and endothelial cells, it is possible to detect the effect of cCPE on living cells via measuring the TJ integrity. Disruption of TJ integrity due to formation of cCPE–claudin complexes can be registered via measuring the transepithelial electrical resistance (TEER) of a monolayer of claudin-expressing cells. Additionally, detection of permeation of labeled molecules, e.g., fluorescein, through the monolayer, is used [37,60,64,65]. This method was, for instance, used to show that GST-tagged cCPE increases the paracellular permeability of fluorescein through the monolayer of Caco-2 cells compared with GST itself [37]. 

Permeation of labeled molecules, e.g., fluorescein or fluorescein-isothiocyanate-dextran (FD), across cellular sheets after application of cCPE-constructs can be detected also *in vivo*. For such assays, the jejunal loop of rats is used most commonly. During the experiment, the lumen of the jejunum is washed with saline and the jejunal loop is prepared by closing both ends with sutures, then a mixture of FD and cCPE is administered into the jejunal loop. cCPE alters the integrity of TJ in epithelial cells and, thereby, molecules of FD can reach the blood by crossing the epithelium paracellularly. At several time points thereafter, blood is collected from the jugular vein and plasma concentration of FD is determined with fluorescence spectrophotometer [59,60,65]. 

### 5.2. Residues in CPE involved in binding to claudins

All methods listed in Chapter 5.1 are used to investigate CPE-claudin interaction. As mentioned in Chapter 3.2, it was shown that the 30 C-terminal amino acids of CPE (β8-loop-β9-region) are essential for binding to claudins. Involvement of residues within this region in binding to claudins has been demonstrated in several studies [35,59,60,63,66]. For instance, it was found that upon deletion of 16 C-terminal amino acids, cCPE loses its ability to interact with Cld4 [67]. The substitution Y310C reduced binding of CPE to brush border membrane in rabbit intestine [35]. Harada *et al.* revealed inhibition of binding and toxicity of cCPE-PSIF [63] in competition assays by substitutions Y306A, Y310A or Y312A [59]. Additionally, this study applied a pull-down assay to show that single mutations Y306A, Y310A or Y312A and double mutation Y310A/Y312A resulted in partial reduction of Cld4 binding. Furthermore, double mutants Y306A/Y310A and Y306A/Y312A, as well as triple mutant Y306A/Y310A/Y312A, lost the ability to bind to Cld4. Measurements of transepithelial resistance of cultured cells and *in situ* loop assays showed inhibition of TJ-opening by the multiple tyrosine mutations [59]. Y306K, but not Y306F substitution interfered with claudin binding [66]. Taken together, these results showed that Y306 is the pivotal residue for binding to claudins, where the aromatic ring and not the functional hydroxyl group is essential. However, all three tyrosine residues (Y306, Y310 and Y312) contribute to the interaction of cCPE with Cld4 and to the modulation of TJ barriers [59]. 

Systematic analysis of each of the last 16 C-terminal amino acids was performed to reveal other residues possibly involved in binding to claudins [60]. By replacing individual amino acids with alanine it was found that L315, in addition to the previously reported tyrosines, is critical for binding to Cld4, modulating TJ-barrier function and enhancing jejunal absorption. The crystal structure of CPE_194–319_ revealed that Y306, Y310, Y312 and L315 form a hydrophobic pit on the surface of CPE, which is obviously involved in claudin binding [37,38]. 

### 5.3. Residues in claudins involved in interaction with CPE

Soon after the identification of Cld3 and Cld4 as CPE-receptors it was demonstrated that CPE does not bind to all claudin subtypes. In particular, binding to Cld3 and Cld4 but not to Cld1 and Cld2 was reported [4]. The association constants for the binding of CPE to Cld3, -4, -6, -7, -8 and -14 were determined to be 8.4 × 10^7^ M^-1^, 1.1 × 10^8^ M^-1^, 9.7 × 10^7^ M^-1^, 8.8 × 10^7^ M^-1^, 1 × 10^6^ M^-1^ and 1 × 10^6^ M^-1^, respectively [4,43]. Recently, detectable binding to Cld1, Cld2 [44] and Cld9 [37] has been demonstrated also. However, CPE-binding to Cld1 and -2 is much weaker than that to Cld3 and -4. For Cld5 no interaction with either full length CPE or CPE_194–319_ was obtained [37]. However, binding of CPE to Cld5 was detected with GST-CPE_116–319_ [37] containing more amino acids than the well described claudin-binding domain (194–319). It was suggested that the very weak affinity between cCPE and Cld5 is presumably enhanced by multivalent binding of GST-CPE_116–319_-oligomers [37]. Different results concerning CPE-binding of the diverse claudin subtypes could be obtained due to differences in the CPE-constructs and the sensitivity of the assay used. However, Cld3, -4, -6, -7, -8 and -9 (controversial for Cld8) can be considered to be high affinity CPE-receptors; Cld1, -2, -5, -8 and -14 (controversial for Cld5 and Cld8) to be low affinity CPE-receptors and Cld10–13 as well as Cld15–24 apparently do not interact with CPE. 

To date, it is well known that the ECL2 of CPE-sensitive claudins is involved in binding with enterotoxin. In different independent studies it was confirmed that CPE interacts with the ECL2 of Cld3 and -4, but not (strongly) with those of Cld1 and -2 [43,62]. In addition, CPE does not recognize ECL1 of Cld3 and -4 [43,44]. Recently, residues in the ECL2 of CPE-binding claudins, involved in the interaction with CPE, were identified by amino acid substitutions in ECL2 of claudins [37]. Arrays with immobilized Cld3-ECL2 peptides were incubated with GST-cCPE. Substitution of D149 and T151 in murine Cld5 by the corresponding residue of Cld3 (N148, L150) enabled the binding of cCPE to Cld5. Similarly, substitution S149N in murine Cld2 led to binding of GST-cCPE. 

Furthermore, substitution mapping was performed to reveal the role of residues in Cld3-ECL2 in interaction with cCPE. Each position of Cld3-ECL2 peptide was substituted with every other amino acid except cysteine. This mapping identified the motif 148NPLVP152 as essential for binding of cCPE to Cld3-ECL2 peptides. Moreover, pull-down and cellular binding assays verified strong involvement of N148 and L150 in interaction between CPE and native Cld3 [37]. Sequence comparison revealed that all cCPE-binding subtypes of claudin contain the consensus sequence NP(L/M)(V/L/T)(P/A) in the turn region of the predicted helix-turn-helix structure of the ECL2 (Chapter 4 and [54]). Strikingly, all claudins, which are able to interact with cCPE, belong to the group of classic claudins [45]. As mentioned above (Chapter 4), the classic claudins probably share a common helix-turn-helix structure. This appears to be a precondition for CPE-binding, since non-classic claudins, which have most likely a different fold, do not bind [37]. However, differences within this helix-turn-helix structure have to determine the ability to interact with cCPE. The positions 2, 4 and 5 in the identified consensus sequence (P, V/L/T and P/A) are highly conserved among classic claudins and, according to the model, are involved in stabilization of the turn region of the loop structure. In contrast, the NH2-group of arparagine at position 1 and side chain of leucine/methionine at position 3 point away from the loop. In all CPE-sensitive claudins, but in nearly none of the CPE-insensitive claudins, asparagine is present at position 1 and leucine or methionine at position 3 of the consensus sequence. Taken together, the discussed data demonstrate that the residues corresponding to N148 and L150 in murine Cld3 are strongly involved in the CPE-claudin interaction.

Subsequently, other studies verified the importance of the asparagine in the proposed consensus sequence. The substitution N149D in full length human Cld4 abrogated the binding of CPE [62]. Conversely, the corresponding D150N substitution in human Cld1 as well as S149N in human Cld2 strongly increased binding of CPE [62]. Kimura *et al.* analyzed several claudin chimera of Cld5 and Cld4 including D149N substitution in human Cld5 [44]. These data are also consistent with conclusions mentioned above.

**Table 1:** Capacity of claudin subtypes and mutants thereof to interact with CPE-constructs. For the claudin constructs the nomenclature of the respective source is given. The respective sequence is given in the left column. It corresponds to W139-W169 of human Cld1 and contains the predicted ECL2, W139-Q163 [45]. Amino acids which differ from the corresponding amino acids in Cld3 and Cld4 (high affine receptors) are highlighted in red. Color code for binding capacity: 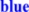
**: Assays based on peptides of claudins or CPE**; 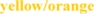
**: assays based on detergent-solubilized claudins**; 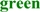
**: cellular-binding assays**. The color shade corresponds to the following categories of binding capacity: 0–10% (-), 10–25% (low), 25–85% (medium) and >85% (high) of bound CPE relative to Cld3wt or Cld4wt. Entries marked with an asterisk were derived by cytotoxicity assays and the categories are defined as: highly sensitive (high), EC50 < 1 µg/mL; slightly sensitive (low), 1 µg/mL < EC50 < 30 µg/mL; and insensitive, 30 µg/mL < EC50 [43]. In some cases, no further quantification of the amount of bound CPE was available (+). All constructs without prefixes are derived from mouse sequences, the other constructs are derived from human (hu-); monkey (mk-); mouse/human chimaeras (muh-). monkey/human chimeras (mkh-). (p) indicates data obtained with CPE_290–319_, (L) data obtained with CPE_116–319_; all other data were obtained with full length CPE or constructs similar to CPE_194–319_.

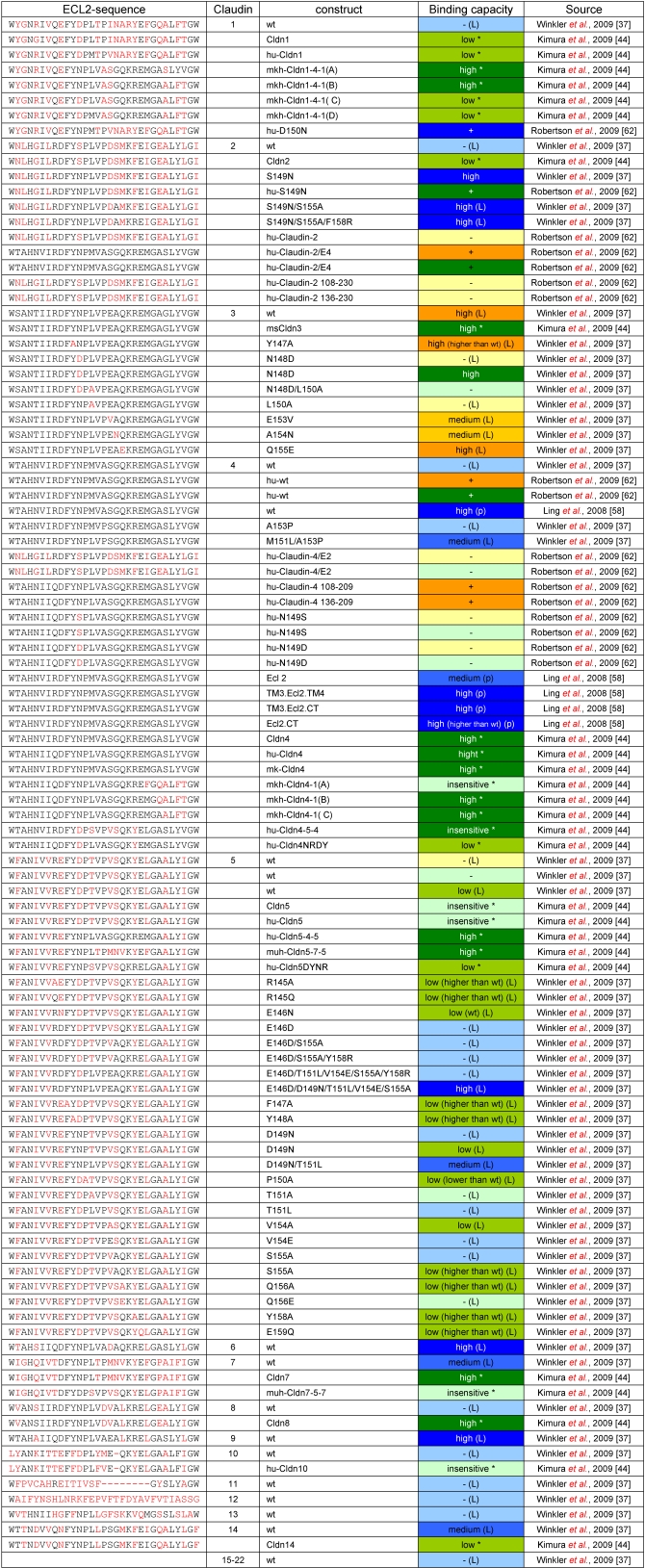

In addition, Kimura *et al.* reported that a Cld4/Cld1 chimera bound to cCPE as long as they contained the particular residue M160 from Cld4 at the end of ECL2, but not the corresponding F161 from Cld1 [44]. In another study, substitution mapping of Cld3-ECL2 peptides [37] suggested that this methionine, which is conserved between Cld4 and Cld3, is rather not directly involved in interaction with CPE. Alternatively, it could change the structure, especially at the transition between ECL2 and the 4th transmembrane segment (TM4) of Cld3/-4 compared to Cld1 and, therefore, play a role in CPE-binding. Results from SRP measurements using recombinant Cld4 protein as analyte are in line with this interpretation. So it was found that CPE_290–319_ binds much stronger to Cld4-constructs containing the TM4 in addition to the ECL2 than the ECL2 alone [58]. Similarly, a recombinant Cld4 construct containing ECL2 and TM4 was able to compete for binding of full length CPE in cellular binding assays [62]. 

Aromatic amino acids in CPE (Y306, Y310 and Y312) are involved in the binding to claudins [60] and aromatic residues in the ECL2 of classic claudin are crucial for *trans*-interaction between each other [54]. It was discussed that the aromatic residues in claudin could also interact with the aromatic amino acids in CPE [58]. But removal of aromatic residues in the ECL2 of Cld3, Cld4 or Cld5 (binding to CPE_116-319_) did not reduce the ability of CPE to bind to its receptors [37,44]. Moreover, in a preliminary model, the aromatic residues in the ECL2 do rather not sterically match the aromatic residues in CPE (Figure 1b and [37]). Taken together, the published data (summarized in Table 1) indicate that residues in the turn region of the loop, but not aromatic residues in the flanking helices, are involved in the claudin-CPE interaction. Residues in the C-terminal flank of the loop and in the following TM4 could contribute to the interaction with CPE either by direct interaction or by determining the structural conformation of the loop. 

It was observed that cCPE binds preferentially to claudin molecules outside of tight junction strands [37]. This is consistent with the idea that CPE sequesters claudins in the plasma membrane, preventing their incorporation in TJ strands. Such a sequestering mechanism could explain the long incubation time with cCPE needed for the opening of TJ, despite the high affinity of cCPE to claudins [43]. 

## 6. Potential Pharmacological Use of *Clostridium perfringens* Enterotoxin

### 6.1. cCPE as a TJ-Modulator

Many promising drug candidates cannot be used clinically because of unacceptable pharmacokinetics. The competence to cross tissue barriers is a major determinant for the delivery of a drug to its target. Several approaches have been used to enhance transcellular drug delivery, including utilization of endogenous influx transporters, blocking of the barrier supporting activity of efflux transporters, or receptor-mediated transcytosis [68]. An alternative strategy tries to enhance paracellular permeation of drugs by transient opening of the TJ [69,70]. The strength of this approach is *(i)* the improvement of the delivery of structurally unrelated drugs and *(ii)* that the drug itself does not have to be modified. Most of the different TJ modulators, described within last years, have surfactant- or chelator-like characteristics. They are often compromised by low tissue specificity and severe side effects, e.g., exfoliation of cells, which irreversibly compromise the barrier functions [71,72]. It was suggested that fewer side effects may be obtained by more specific modulation of a molecular key component of the TJ [73]. cCPE is a promising tool to modulate TJ in a direct and tissue-specific manner: (1) It binds with high affinity to claudins that are essential for TJ formation. (2) CPE binds to a subset of claudins, only. This restricts its activity to tissues that express CPE-sensitive claudins. (3) It is able to increase the paracellular permeability in a reversible and concentration-dependent manner. (4) cCPE enhances drug absorption in rat jejunum 400-fold relative to sodium caprate, which is in clinical use [5]. In addition, cCPE is known to enable mucosal absorption of a biologically active peptide [65]. Cld1 and Cld5 are potential targets for transepidermal and brain drug delivery, respectively [74,75]. However, it has been reported that these claudins do not strongly interact with CPE [43]. Alternatively, Cld3 could be considered to improve the brain uptake. Moreover, modification of cCPE could shift its claudin-subtype specificity to enable drug delivery to the brain.

### 6.2. Treatment of tumors overexpressing claudins by cCPE-constructs

Several studies have suggested the use of CPE for the chemotherapy of tumors overexpressing claudins [76,77,78]. The CPE-receptors Cld3 and Cld4 are strongly upregulated in many pancreatic, ovarian, breast and uterine cancers [76,79]. The use of other surface proteins for targeting cancer cells has been already demonstrated. For instance, ligands for cytokine- and growth factor receptors have been fused with fragments of bacterial toxins, such as *diphtheria* toxin and *Pseudomonas* exotoxin [80,81]. Similarly, fusion proteins of *diphtheria* toxin fragment A and cCPE were generated to target Cld4-overexpressing tumor cells [82]. CPE-fragments fused to tumor necrosis factor effected human ovarian cancer cells [83]. Injections of full length and hence cytotoxic CPE into pancreatic tumors in mice resulted in tumor necrosis and reduction of tumor growth [84]. Human ovarian cancer cells transplanted in mice could be also eliminated by injection of CPE [85]. Intracranial CPE administration reduced growth of breast cancer brain metastasis and increased survival in murine models [76]. Recently, cCPE fusion proteins were successfully used for nasal vaccination, a strategy that could be used for different therapeutic approaches including tumor treatment [82]. These studies demonstrated the therapeutic potential of CPE-constructs.

## 7. Conclusions and Remarks

In the last decade tremendous progress has been made in the characterization of the structure and function of CPE and its receptors, the claudins. Of particular importance is the solved crystal structure of the claudin-binding domain of CPE and recent advances in identification of residues in CPE and claudins that are involved in the interaction between each other. Claudins are highly relevant for tissue barriers, tumor growth and, as recently discovered, hepatitis virus C infection [86]. CPE has already successfully been applied to target claudins for therapeutic approaches in animal models. Hence, future progress in the molecular understanding of the CPE-claudin interaction may facilitate the development of improved recombinant cCPE proteins and cCPE-based peptidomimetics as powerful pharmacological tools.
